# The Promoting Resilience in Stress Management (PRISM) intervention for adolescents and young adults receiving hematopoietic cell transplantation: a randomized controlled trial protocol

**DOI:** 10.1186/s12904-022-00966-9

**Published:** 2022-05-19

**Authors:** Kaitlyn M. Fladeboe, Samantha Scott, Liam Comiskey, Chuan Zhou, Joyce P. Yi-Frazier, Abby R. Rosenberg

**Affiliations:** 1 Department of Pediatrics, Division of Hematology/Oncology, University of Washington School of Medicine, Seattle, WA USA; 2 Palliative Care and Resilience Lab, Center for Clinical & Translational Research, Seattle Children’s Research Institute, M/S CURE-4, PO Box 5371, Seattle, WA 98145-5005 USA; 3 Department of Psychology, University of Denver, Denver, CO USA; 4 Department of Pediatrics, Division of General Pediatrics, University of Washington School of Medicine, Seattle, WA USA; 5 Center for Child Health, Behavior and Development, Seattle Children’s Research Institute, Seattle, WA USA; 6 Cambia Palliative Care Center of Excellence, University of Washington School of Medicine, Seattle, WA USA

**Keywords:** AYA, Pediatrics, Childhood, Cancer, Hematopoietic cell transplant, Psychosocial outcomes, Mental health, Quality of life, Resilience, Palliative care

## Abstract

**Background:**

Psychological distress is prevalent among adolescents and young adults (AYAs) receiving hematopoietic cell transplantation (HCT). The Promoting Resilience in Stress Management (PRISM) intervention is a resilience-coaching program that has been shown to mitigate distress and improve quality of life among AYAs receiving chemotherapy for newly diagnosed or advanced cancer. This article describes the protocol of an ongoing randomized-controlled trial (RCT) examining the efficacy of PRISM among AYAs receiving HCT for cancer and/or blood disorders.

**Methods/design:**

The goal of this multi-site, parallel, RCT is to evaluate the effect of PRISM compared to psychosocial usual care (UC) among AYAs receiving HCT. Our primary hypothesis is that AYAs who receive PRISM will report lower depression and anxiety 6-months following enrollment compared to those who receive UC. The PRISM program includes four scripted coaching sessions targeting skills in stress-management, goal setting, cognitive-restructuring, and meaning-making, followed by a facilitated family meeting. Sessions are delivered one on one, 1–2 weeks apart, in-person or via videoconference. We aim to recruit 90 AYAs from 4 US pediatric AYA oncology centers. Eligible AYAs are aged 12–24 years; receiving HCT for malignancy or a bone marrow failure syndrome associated with cancer predisposition; < 4 weeks from their HCT date; able to speak English and read in English or Spanish; and cognitively able to complete sessions. Enrolled AYAs are randomized 1:1 within each site to receive PRISM+UC or UC alone. AYAs on both study-arms complete patient-reported outcome surveys at baseline, 3- and 6-months. Age-valid instruments assess depression and anxiety, overall and cancer-specific health-related quality of life, symptom burden, resilience, and hope. Covariate-adjusted regression models will compare AYA-reported depression and anxiety at 6-months in the PRISM versus UC groups. Secondary and exploratory objectives include assessments of PRISM’s cost-effectiveness and its impact on (i) parent and caregiver quality of life and mental health, (ii) pharmaco-adherence to oral graft-versus-host disease (GVHD) prophylaxis, (iii) biologic outcomes such as transplant engraftment and graft-versus-host disease, and (iv) biomarkers of stress such as heart rate variability and the Conserved Transcriptional Response to Adversity (CTRA) gene expression profile.

**Discussion:**

If successful, this study has the potential to address a critical gap in whole-patient care for AYAs receiving HCT.

**Trial registration:**

ClinicalTrials.gov Identifier NCT03640325, August 21, 2018.

## Background

The experience of hematopoietic cell transplantation (HCT) among Adolescents and Young Adults (AYAs) with cancer is particularly difficult because age-related developmental challenges of identity, relationships, and vocation may add to the burden of cancer. AYAs receiving HCT experience high burdens of medical morbidity and mortality, and comparatively poor health-related quality of life [1–8]. Anxiety is highly prevalent before the transplant [9], and 40% of patients experience depressive symptoms in the 6 months that follow [10, 11]. This “stress reaction” is associated with behavioral problems and peer isolation, and persists within a third of patients [10]. A potential barrier to improving these experiences is that AYAs have few opportunities to develop the personal resilience resources needed to navigate such adversity.

Helping AYAs develop these resources early in the transplant period may minimize escalation to significant distress. However, despite national recommendations for early integration of psychosocial care in pediatric and AYA oncology [12–17], access to early psychosocial interventions is still limited. We previously developed the “Promoting Resilience in Stress Management” (PRISM) intervention for AYAs with serious illness [18]. This manualized, brief intervention targets skills in stress-management and mindfulness, goal-setting, positive reframing, and meaning-making. These skills have been shown to be associated with improved patient well-being in other populations [19–21] and findings from a phase 2 randomized controlled trial among AYAs with newly diagnosed or advanced cancer suggested PRISM improved psychological distress and health-related quality of life compared to usual non-directive supportive care [22–24]. Thus, PRISM may be similarly helpful in mitigating psychological distress among AYAs receiving HCT. This article describes the protocol of an ongoing multi-site randomized-controlled trial aiming to evaluate the impact of PRISM among AYAs receiving HCT.

## Methods/design

The primary aim of this study is to evaluate the effect of PRISM compared to usual care (UC) on AYA-reported symptoms of depression and anxiety 6-months post-enrollment. We hypothesize that PRISM will be associated with lower depression and anxiety compared to UC. We additionally aim to evaluate the impact of PRISM on other key patient-reported outcomes 6-months following enrollment, including symptom burden, health-related quality of life, hope, and resilience. We hypothesize that PRISM will be associated with lower total symptom burden; higher quality of life; higher hope; and higher resilience, compared to UC.

Additional secondary and exploratory aims include: (a) to evaluate the cost-effectiveness of the PRISM intervention; (b) to evaluate PRISM’s impact on parent/caregiver health-related quality of life, anxiety and depression symptoms 6-months following enrollment; (c) to evaluate the impact of PRISM on adherence to oral Graft-Versus-Host Disease (GVHD) prophylaxis; and (d) to prospectively describe associations between PRISM, patient reported anxiety and depression, and stress biomarkers including days from HCT to neutrophil engraftment, prevalence and severity of GVHD, heart rate variability, and the Conserved Transcriptional Response to Adversity (CTRA) gene expression profile.

### Design, setting, and participants

This is a multi-site, phase 3, parallel, 1:1 randomized controlled trial conducted at four academic, pediatric AYA oncology centers in the United States (Table 1). Participating sites include the Seattle Cancer Care Alliance (SCCA; Seattle Children’s Hospital and Fred Hutchinson Cancer Research Center), Children’s Hospital of Los Angeles (CHLA), the University of Alabama at Birmingham (UAB) Children’s Hospital, and St. Jude Children’s Research Hospital (SJCRH). Participants are randomized 1:1 to receive psychosocial UC or PRISM+UC. To be eligible, AYAs must be aged 12–24 years; receiving allogeneic or autologous HCT for treatment of malignancy or bone marrow failure syndrome with cancer predisposition; within 4 weeks of their HCT date at the time of consent; able to speak in English; able to read in English or Spanish; and cognitively able to participate in the intervention and complete surveys.

**Table 1:**
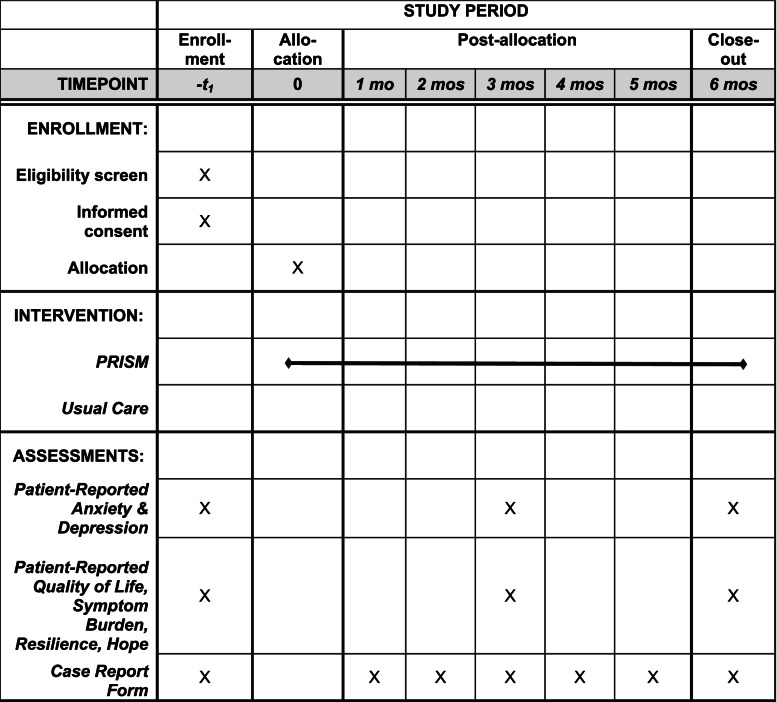
Schedule of enrollment, PRISM intervention, and assessments

### Recruitment and informed consent

Potentially eligible AYAs are recruited from outpatient clinics and inpatient wards. Study staff screen patients via review of the new transplant “arrivals” list, clinic rosters, clinician communications, and inpatient census reports followed by a review of the electronic health record to verify eligibility. Eligible AYAs and their families are introduced to the study through a study information flyer and/or opt-out letter. Consent conferences are conducted in clinic, inpatient rooms, or via phone or videoconference when in-person recruitment is not possible/feasible (e.g., due to COVID-19 visitor restrictions). For all procedures, AYAs provide written assent (if aged 12–17 years) or consent (if aged 18–24 years), and parents provide written consent (for patients aged 12–17 years).

### Randomization

Following provision of consent and completion of baseline surveys, AYAs are randomized 1:1 to usual, non-directive, supportive care (UC, “control”) or PRISM+UC (“intervention”) within strata defined by age group (AYAs ages 12–17 versus 18–24) and study site. The study statistician constructed the randomization algorithm using a permuted blocks scheme with randomly varying block sizes. Research associates administer randomized assignments using Research Electronic Data Capture (REDCap). As a quality control measure, a randomization log is maintained to track the participant ID, stratum, randomization assignment, and date of randomization. Biostatisticians who will conduct data analysis are blinded from the treatment group allocations.

### The Promoting Resilience in Stress Management (PRISM) intervention

Participants randomized to the intervention arm receive the Promoting Resilience in Stress Management (PRISM) program. PRISM consists of four scripted, 30–50 minute, one-on-one, in-person coaching sessions delivered approximately 1–2 weeks apart. Each session targets a specific resilience resource (Table 2). PRISM is delivered by research staff (“coaches”) who have a bachelor’s degree or higher education and who have completed PRISM’s certification training [18, 25]. This training includes at least 8 hours of supervised didactics and role-playing scenarios with progressively complicated scenarios, at least 2 supervised program deliveries to demonstrate mastery, and focused training on recognizing distress, screening for risks of self-harm, and procedures for immediate intervention (i.e., contacting suicide hotlines and/or local medical and psychosocial clinicians).

**Table 2:** Promoting Resilience in Stress Management Intervention Content

Session	Skills Taught During Session	Format
1. Managing stress	Mindfulness techniques, relaxation strategies	One on one
2. Goal setting	Setting specific, realistic, desirable goals; planning for roadblocks	
3. Positive reframing	Recognizing negative self-talk; replacing it with realistic, positive, manageable thoughts	
4. Meaning making	Identifying benefits, purpose, meaning, or legacy from cancer experience	
5. Coming together	Discussion about what was learned, what helped, what loved ones can do to help	Family meeting
6. Boosters	Check-in visits to practice, further develop, and track skills	One on one
7. Cheat sheets	Between-session exercises to practice, further develop, and track skills	Paper and pencil or PRISM App

Sessions are delivered approximately every 1–2 weeks, arranged in advance in conjunction with patient clinical and hospital visits. Sessions 5 & 6 are offered to all participants as optional opportunities for continuing to practice and/or share skills

Sessions are scheduled around AYAs’ clinic and/or hospital visits (depending on concurrent illness and medical needs). Following the final session, PRISM-AYAs are offered monthly “booster” contacts until they reach the 6-month endpoint. Although in person visits are preferred, patients may request sessions and boosters via phone or other web-based communication (i.e., zoom, WebEx) if scheduling barriers preclude in-person visits. Sessions are discontinued if illness or death precludes participation or if requested by the participant. Participants who request to discontinue PRISM sessions may still complete follow-up survey assessments. *S*essions are audio-recorded and scored for fidelity using a standardized tool by a supervising licensed clinical psychologist. Coaches receive biweekly 1:1 supervision, which includes feedback and, if necessary, re-training to address fidelity concerns.

Details of the sessions are listed in Table 2. Briefly, Session 1 (“Stress Management”) focuses on relaxation skills including deep breathing and guided imagery, and mindfulness techniques including an exercise to become aware of stressors and powerful emotions without judgement. Session 2 (“Goal Setting”) teaches “SMART” goal-setting skills (e.g., identifying specific, measurable, actionable, realistic, and time-dependent goals, planning steps towards their achievement, and preparing for roadblocks). Session 3 (“Cognitive Restructuring”) trains participants to recognize negative emotions and demoralizing self-talk and then develop skills to reframe these in a positive light. Session 4 (“Benefit Finding”) focuses on finding meaning and/or benefitting from difficult situations, including cancer. The final 5th session (“Coming Together”) allows patients to reflect on the skills they have learned and share their perspectives and experiences with their parent(s), caregiver(s), spouse, or significant others. For families where family members or others prefer Spanish or another non-English language, we conduct the final session with a certified interpreter of the native language of the parent. Finally, to practice skills between sessions, all participants receive paper-pencil worksheets reviewing each PRISM skill and are invited to download the PRISM mobile app to practice the skills on their smartphone. Briefly, the app mimics the paper-pencil worksheets with 6 digital worksheets that may be logged within the program. The stress-management modules include audio-recordings of the relaxation and mindfulness instructions. The goal setting, cognitive restructuring, and meaning-making modules include direct-entry text-based “journaling” activities. The app also contains tracking systems for AYAs to note their own perceptions of stress and resilience, a calendar function to track their goals (including task reminders), and a photo-journal to catalogue goals, re-structured thoughts, and gratitudes.

### Assessments

#### Medical record abstraction

Trained study staff complete monthly electronic health record abstraction via a Case Report Form (CRF) capturing the following variables: Mental health care utilization and additional relevant health care and service utilization (e.g., number and types of supportive care medications; number of, duration, and reasons for unplanned hospital days, emergency department, and outpatient clinic visits; and resource use associated with additional health problems and comorbidities). Clinical covariates abstracted from the electronic health record also include cancer or marrow failure diagnosis, prior treatment regimens, presence and severity of Graft-Versus-Host Disease, and associated treatments.

#### Patient-reported outcome surveys

At enrollment, 3-months, and 6-months, AYAs on both arms complete a comprehensive survey comprised of age-appropriate validated instruments and standard demographics. Baseline surveys must be completed within 2 weeks of enrollment. Subsequent surveys must be completed within 28 days of their due date. Participants are given weekly reminders via phone, email, or in-person until surveys are completed. Participants are paid $25 for completion of each survey.

Our primary outcome is patient-reported depression and anxiety measured by the Hospital Anxiety and Depression Scale (HADS). The HADS assesses mixed affective symptoms in patients with serious illness [26]. It has been validated in AYAs with chronic illness [27] as well as AYA survivors of cancer [28]. It consists of 7 questions for anxiety and 7 for depression. Each is scored from 0 to 3 and summed for a total score ranging from 0 to 21 points per subscale. Higher scores indicate worse symptoms. Both subscales have excellent reliability (HADS-Anxiety α = 0.83; HADS-Depression α = 0.84) [26]. “Caseness” of depression or anxiety is defined as ≥8 points on either subscale, with sensitivity/specificity of 80%/80% for depression and 80%/90% for anxiety [26].

Secondary outcomes include: (a) Symptom burden, as measured by the Memorial Symptom Assessment Scale (MSAS) [29, 30]. This instrument assesses the presence, severity, frequency, and extent of bother from 26 symptoms [31, 32]. Each symptom is scored from 0 to 4 for severity, frequency, and extent of bother, with higher scores suggesting more severe, frequent, and bothersome symptoms. (b) Quality of Life, as measured by the PedsQL 4.0 Generic and 3.0 Cancer Modules [33]. Queries assess physical, emotional, social, and school well-being, plus cancer-related pain and hurt, nausea, procedural anxiety, treatment anxiety, worry, cognitive problems, perceived physical appearance, and communication. Scores range from 0 to 100, with higher scores suggesting better quality of life. (c) Hope, as measured by the Snyder “Hope” Scale. This instrument contains 8 hope items plus 4 “filler” questions and measures “the overall perception that one’s goals can be met.” [34] Scores range from 8 to 64, with higher scores suggesting more hopeful patterns of thought. (d) Resilience, as measured by the Connor-Davidson Resilience Scale (CD-RISC), a reliable and widely used instrument to measure self-perceived resilience. Scores range from 0 to 40, with higher scores suggesting higher resilience [35, 36].

### Sample size & power

Sample size and power calculation were based on primary outcome HADS score at 6-months. Preliminary data from our Phase 2 trial showed AYA receiving PRISM had HADS scores normally distributed with mean score of 11.1 (SD = 6.2). Assuming 20% attrition, we will randomize 90 AYAs (45 per arm) to obtain a final sample size of 70 AYA participants (35 per arm) with 6-month data. This sample size achieves 80% power to detect a 4.2-point difference in the mean 6-month total HADS score between PRISM and UC arms at 0.05 type I error rate.

### Data analysis

The main analytic approach for primary and secondary analyses will be covariate-adjusted linear regression models. For the primary analysis, the total HADS score will be the outcome, the PRISM intervention indicator will be the predictor of interest, and baseline HADS score, age group, and site will be adjusted as covariates in the regression. With this model specification, the regression coefficient of the PRISM indicator captures the difference in the average changes (from baseline to 6 months) for the HADS score between the PRISM arm and usual care arm. Thus, the primary hypothesis can be tested by applying the Wald t-test to the regression coefficient of the PRISM indicator. The same analysis will be undertaken for the domain subscales of anxiety and depression (HADS-A and HADS-D subscale scores) and for secondary outcomes (symptom burden, resilience, hope, and health-related quality of life). Multiple comparisons are a concern as we are collecting multiple measures from patients and are interested in several hypotheses. We minimize this problem by specifying a limited number of main hypotheses for each aim. The Benjamini-Hochberg procedure will be used to control the False Discovery Rate criterion at 0.05 level to correct for multiple testing in analyses that are not pre-specified [37].

To minimize missing data, surveys are reviewed within 72 hours for missingness. However, data may still be missing due to participants skipping individual survey items, omissions in electronic health records, lack of follow-up, medical and psychosocial complications, or death. We will quantify the amount of missing data, evaluate the pattern of missingness and association of participant characteristics with missing data, and minimize bias and increase efficiency in the associations of interest by applying appropriate methods to account for missing data [38–40]. Given the RCT design, we anticipate missing data will mostly occur in outcomes. For outcomes where missing at random (MAR) is a plausible assumption, we will apply multiple imputation techniques to address the missing data problem. However, for this patient population (AYAs with cancer) and the types of our intended outcomes (mental health, psychosocial, behavioral), missing data tend to be linked to skipped surveys, missed visits, or death, which makes missing not at random (MNAR) a more plausible scenario. Given this, we will conduct sensitivity analysis using controlled *δ*-based multiple imputation techniques [41]. In δ-based imputation, an offset term, δ, is typically added to the expected value of the missing data to assess the impact of unobserved participants having a worse or better response than those observed [42].

### Data Safety & Monitoring

Study clinical and data coordination is based at the primary site (Seattle Children’s Hospital and the Seattle Cancer Care Alliance). The study PI supervises all study activities, with the support of all co-investigators and site PIs. The primary site study staff include a lead staff supervisor with extensive experience in clinical trial regulations and procedures, including data-collection and management; a lead clinical research coordinator who is responsible for ongoing project oversight, including ongoing monitoring of enrollment, data-completion, and site-level troubleshooting; and a lead PRISM coach who is responsible for coach-training and supervision. The primary site staff also includes a Master’s-level biostatistician who is blinded to intervention arm and supports regular data-entry and cleaning, plus a PhD-level biostatistician who directs analytic plans and considerations for data-interpretation. Due to the nature of the intervention, clinical PIs and site-staff are not blinded to randomization-assignment.

The full study team (primary site staff, including PI, Co-Is, and statisticians, plus all site PIs and their site-specific staff) meets monthly to review study progress, data completion, and regulatory needs. The SCH lead clinical research coordinator meets with research coordinators from each site twice monthly to review and troubleshoot trial conduct and questions, including regulatory oversight, recruitment, data collection, intervention delivery, clinical concerns and/or other concerns. To ensure data quality, a study staff member reviews all surveys within 72 hours for completeness and contacts participants to query/complete individual missing items verbally. Quality of abstracted electronic health record data is ensured via training protocols (i.e., guided practice abstraction and independent abstraction with reconciliation by a trainer) and dual abstraction for 10% of CRFs. The Seattle Children’s Hospital lead coach meets with coaches from all sites twice monthly to review and trouble-shoot intervention session challenges and review other questions that arise in real time. As above, PRISM-fidelity is monitored by the supervising PRISM coach using a standardized tool; individual coaches also meet with the lead coach, as needed, to review their scores and receive additional training.

Data monitoring occurs in real time within the REDCap system. Prior to site closure and corresponding database-lock, all sites will also be monitored by the primary supervisor and lead clinical research coordinator, with the goal of verifying source data.

Data safety monitoring is conducted by a 4-member Data Safety Monitoring Committee (DSMC) independent of the protocol. The committee is convened twice annually to provide input and guidance on the study evaluation and intervention protocols and data handling activities. DSMC members provide input and feedback to the PI and co-investigators related to (a) accrual rate, (b) study eligibility determination issues, (c) data completion rates including conformance with informed consent requirements, (d) intervention fidelity indicators, (e) adverse events, and (f) compliance with data management procedures. This study does not have pre-set stopping rules, but the DSMC has the option of requesting the data be un-blinded and may alter the study or stop the study early.

Adverse events information is collected at all assessment points and recorded on standard forms. Consistent with NIH and site IRBs policies, serious adverse events will be promptly reported in writing to the NIH, the local IRB, and the DSMC chair. The DSMC may modify or stop the study if any such complaint represents a legitimate concern about the study procedures or methods.

### Ethics & Dissemination

All procedures have been approved by the Institutional Review Boards of each participating site. Protocol modifications are first approved by the primary coordinating site (Seattle Children’s Hospital) IRB and then submitted to each site’s local IRB. The lead clinical research coordinator ensures all sites obtain local IRB-approval prior to implementing changes. If needed, any major modifications (e.g., changes to eligibility criteria) will first be approved by the sponsor.

To ensure participant safety, survey data are reviewed within 72 hours for unanticipated immediate threats to participants’ or others safety. As above, PRISM coaches are trained to recognize concerning language during sessions. If concerns arise, the PI or lead interventionist alerts the participants’ primary medical and social works teams for further consultation or referral. If indicated, they may also provide local suicide hotline numbers and/or keep participants online (or on the phone) while contacting the local authorities. As part of the informed consent process, participants are made aware that confidentiality may be broken in the case that study staff see an immediate threat to the patient’s or another’s safety.

To ensure participant confidentiality and privacy, all information collected for research purposes is coded with participant identifiers. The only link between the identifiers and protected health information is stored on a secure database. Individual sites are responsible for original source data until study completion. Minimal paper records are kept in a locked filing cabinets and do not include any identifiable patient information. Data are stored in a HIPAA-compliant electronic database (REDCap - Research Electronic Data Capture) using the participant’s study identifier. Study investigators will have sole access to the final dataset. Coded (de-identified) study data will be banked indefinitely, and access will be controlled the PI. De-identified data may be available to other investigators based on written request to the PI. Trial results with be disseminated to the scientific community via manuscript publications, presentations at scientific meetings, podcasts, social media, and other formal presentations.

## Discussion

This paper describes the protocol for a multi-site RCT of the Promoting Resilience in Stress Management (PRISM) intervention for AYAs receiving hematopoietic cell transplantation (HCT). PRISM is a skills-based training program in which recipients learn to develop and apply key resilience skills during challenging circumstances. The goal of this study is to examine whether delivering PRISM early in the HCT experience is associated with improved psychosocial outcomes compared to usual care 6-months later.

This study has several important strengths. First, few evidence-based psychosocial supportive care programs exist for AYAs undergoing transplant despite their known risks for poor outcomes [10]. Given PRISM’s prior success among AYAs with serious illness [22–24, 43], this study represents an important step toward addressing this gap. Second, this trial includes four sites, each representing geographically and demographically unique areas of the United States. This will facilitate analyses of a more diverse and representative sample than prior PRISM studies and, thus, enhance generalizability of findings. Third, the project includes innovative exploratory aims to assess PRISM’s cost-effectiveness, impact on adherence health behaviors, impact on parent/caregiver outcomes, and relationship to biomarkers of stress and clinically relevant biologic outcomes. It will thus provide key foundational data regarding the role programs like PRISM can play in larger health care and family systems.

Relevant limitations of this protocol should also be noted. First, all sites in this trial are academic, pediatric-based AYA oncology centers; findings may not generalize to community-based or adult centers. Second, inclusion criteria may also limit generalizability as AYAs must be proficient in spoken English to participate. Though beyond the scope of the current study, translation and validation of PRISM in languages other than English is an ongoing and important next step. Third, this study does not include an active (“attention”) control. Thus, we will not be able to discern to what extent intervention effects are attributable to PRISM’s content versus social engagement with coaches. Fourth, as the study endpoint is 6-months post-enrollment, we will not be able to assess the durability of intervention effects beyond this window.

Finally, conducting psychosocial research with this medically complex population involves inherent challenges. We aim to enroll AYAs early in the HCT experience to examine whether PRISM may mitigate immediate post-transplant distress because this is the period associated with the highest risks of poor mental health. However, this period is highly stressful for patients and families, which may impact willingness to participate in research. In addition, though PRISM is designed to be delivered in a brief and flexible manner to accommodate patient needs, PRISM session and survey completion may be hindered by treatment sequelae or complications. Similarly, this study is being conducted during the COVID-19 global pandemic. Participants may be experiencing additional stressors that preclude their enrollment and/or completion of the study. Those stressors also may influence PRISM’s efficacy.

Given high rates of psychological distress among AYAs during and after HCT, empirically supported psychosocial programs are critically needed. PRISM is a brief, manualized program with a growing evidence base designed to help AYAs with serious illness develop and apply resilience resources during stressful periods of their treatment. If receiving PRISM is associated with a reduction in post-HCT distress as hypothesized, this will represent an important advance toward mitigating long-term negative outcomes for this population.

## Data Availability

The datasets generated and/or analyzed during the current study are not publicly available due to participant privacy concerns but are available from the corresponding author on reasonable request.

## Abbreviations

AYA: Adolescent and young adult
CTRA: Conserved Transcriptional Response to Adversity
DSMC: Data safety and monitoring committee
HADS: Hospital Anxiety Depression Scale
HCT: Hematopoietic cell transplant
NIH: National Institutes of Health
PRISM: Promoting Resilience in Stress Management
RCT: Randomized controlled trial
REDCap: Research Electronic Data Capture
UC: Usual care
